# Insulator-to-half metal transition and enhancement of structural distortions in $\text {Lu}_2 \text {NiIrO}_6$ double perovskite oxide via hole-doping

**DOI:** 10.1038/s41598-020-80265-6

**Published:** 2021-01-13

**Authors:** Safdar Nazir

**Affiliations:** Department of Physics, University of Sargodha, Sargodha, 40100 Pakistan

**Keywords:** Materials science, Condensed-matter physics

## Abstract

Using density functional theory calculations, we found that recently high-pressure synthesized double perovskite oxide $\text {Lu}_2 \text {NiIrO}_6$ exhibits ferrimagnetic (FiM) Mott-insulating state having an energy band gap of 0.20 eV which confirms the experimental observations (Feng et al. in Inorg Chem 58:397–404, 2019). Strong antiferromagnetic superexchange interactions between high-energy half-filled $\text {Ni}^{+2}$-$e_g^2\uparrow$ and low-energy partially filled $\text {Ir}^{+4}\,t_{2g}^3\uparrow t_{2g}^2\downarrow$ orbitals, results in a FiM spin ordering. Besides, the effect of 3*d* transition metal (TM = Cr, Mn, and Fe) doping with 50% concentration at Ni sites on its electronic and magnetic properties is explored. It is established that smaller size cation-doping at the B site enhances the structural distortion, which further gives strength to the FiM ordering temperature. Interestingly, our results revealed that all TM-doped structures exhibit an electronic transition from Mott-insulating to a half-metallic state with effective integral spin moments. The admixture of Ir 5*d* orbitals in the spin-majority channel are mainly responsible for conductivity, while the spin minority channel remains an insulator. Surprisingly, a substantial reduction and enhancement of spin moment are found on non-equivalent Ir and oxygen ions, respectively. This leads the Ir ion in a mixed-valence state of $+4$ and $+5$ in all doped systems having configurations of $5d^5$ ($t_{2g}^3\uparrow t_{2g}^2\downarrow$) and $5d^4$ ($t_{2g}^2\uparrow t_{2g}^2\downarrow$), respectively. Hence, the present work proposes that doping engineering with suitable impurity elements could be an effective way to tailor the physical properties of the materials for their technological potential utilization in advanced spin devices.

## Introduction

Recently, double perovskite oxides (DPO) having a chemical formula $\text {ABB}^\prime \text {O}_6$ (A = alkaline earth or rare earth metal atoms and $\text {BB}^\prime = 3\textit{d}$ and 4/5*d* transition metals such as B = Fe, Cr, Mn, Co, and Ni; $\text {B}^\prime = \text {Mo}$, Re, Os, Ir, and W) have been attracting a lot of attention due to their unusual physical properties such as large magnetoresistance at or above room temperature^1–6^, high-temperature ferromagnetism(FM)/ferrimagnetism(FiM)^7–10^, half-metallicity^6,11–14^, FM/FiM Mott-insulator^15–18^, multiferroicity^19^, exchange bias^20^, and magneto-dielectricity^21^. Particularly, a half-metallic (HM) state in $\text {Sr}_2 \text {FeMoO}_6$^1,7,22^ and $\text {Sr}_2 \text {FeReO}_6$^2,23,24^ (i.e., one spin channel exhibits a conducting nature while the other is insulator or semiconductor) having Curie temperature ($T_C$) of 400–415 K is found, which displayed novel applications for spintronics perspective^25,26^. In most of the experiments, it is analyzed that HM is FM such as $\text {La}_{1-x} \text {Sr}_x \text {MnO}_3$^27^ and few are HM FiMs or highly spin-polarized like $\text {Sr}_2 \text {CrReO}_6$ having a $T_C$ of 635 K^8,9,28,29^. The highest $T_C$ of 725 K is observed in $\text {Sr}_2 \text {CrOsO}_6$ with the magnetic moment of 1.92 to 2.04 $\mu _B$^12,15,16^. Very recently, Feng et al.^18^, synthesized disordered monoclinic $\text {Lu}_2 \text {NiIrO}_6$ (LNIO) DPO under high-pressure (6 GPa) and high-temperature (1300 °C), where authors observed a FiM Mott-insulating state having a highest $T_C$ of 207 K among the disordered DPOs. A FiM Mott-insulating ground state in the LNIO was also verified by first-principle calculations, where a strong superexchange AFM coupling between Ni and Ir ions is energetically favorable^30^. A small and large energy band gaps ($E_g$) of 0.20 and 2.25 eV exist in the spin-majority and spin-minority channels, respectively. Hence, due to a small energy gap and high $T_C$ among the Ir-based DPOs, LNIO could be an essential material in the designing of hard magnetic memory devices by tailoring its properties. The above mention properties of the DPOs have been stimulated the researchers to synthesize or predict new materials with improved electronic and magnetic properties.

As it is experimentally and theoretically established that DPOs are considered beneficial candidates for electron and hole-doping at A or B site, aiming to obtain optimized physical properties that were not present in their undoped form^31–47^. For example, in a widely studied $\text {Sr}_2 \text {FeMoO}_6$ DPO, electron doping which is obtained by partial replacement of $\text {Sr}^{+2}$ with $\text {La}^{+3}/\text {Nd}^{+3}$ results in the enhancement of $T_C$^31–34^. Interestingly, the electrical resistivity of the $\text {Sr}_2 \text {FeMoO}_6$ system decreases when a Sulfur ion is doped at oxygen site and FiM or AFM spin ordering-like behavior arise at low temperatures^41^. Similarly, Geprags et al.^35^, experimentally observed that electron doping in the $\text {La}_2 \text {CrWO}_6$ FiM DPO usually enhances the $T_C$. Blasco et al.^37^, experimentally observed that magnetic transition temperature increases with the increase of La doping at Sr site in $\text {Sr}_2 \text {CrMoO}_6$. Along with this, $\text {La}^{+3}$ doping at $\text {Ca}^{+2}$ site in $\text {Ca}_2 \text {FeIrO}_6$ results in the modulation of microscopic magnetic coupling, which further leads to the magnetic phase transition from AFM to FiM^36^. Similarly, an AFM to FiM spin ordering transition is observed in $\text {(Ca,Sr)}_2 \text {FeIrO}_2$ system due to $\text {La}^{+3}$-doping and authors claimed that changes in the microscopic magnetic interaction may be associated with the mixed-valence state of Ir^38^. In a similar fashion, $\text {Pr}_{2-x} \text {Sr}_x \text {MgIrO}_6$ system exhibits a transition from Mott-insulating to HM AFM state at $\textit{x} = 1.0$, where Ir-ion remains in mixed-valence states of $+4$, $+4/+5$, and $+5$ for $\text {Pr}_2 \text {MgIrO}_6$, $\text {Pr}_{1.5} \text {Sr}_{0.5} \text {MgIrO}_6$, and $\text {PrSrMgIrO}_6$ samples, respectively^45^. Moreover, Coutrim et al., experiments show that variations of the magnetic coupling between Co and Ir ions in $\text {La}_2 \text {CoIrO}_6$ are due to a change of their valence, when La is replaced with Ca ion having a concentration of $0\le$Ca $\le 1.2$^40^. It is also observed that net magnetization and $T_C$ decreases when Ca-doped in $\text {La}_2 \text {CoIrO}_6$. However, the magnetization of $\text {Sr}_2 \text {MnMoO}_6$ compound increases at a very low concentration ($\le 0.15$) of Bi doping at Sr site, which also illustrates an AFM coupling between $\text {Mn}^{+2}$ and $\text {Mo}^{+5}$ ions and results in a FiM spin ordering^42^.

From a theoretical point of view, it is found that hole-doping (i.e., $\text {Sr}^{+2}$-doping at $\text {La}^{+3}$ site) in a FiM $\text {La}_2 \text {VMnO}_6$ ($\text {La}_{2-x} \text {Sr}_x \text {VMnO}_6$) leads the system to HM state^39^. It is also demonstrated that holes remain in the V 3*d* orbitals for $\textit{x} = 0.5$ and 1.0 and thenceforth, lie in the Mn 3*d* orbitals for $\textit{x} = 1.5$ and 2.0. Alike, the hole-doping (i.e., $\text {Na}^{+1}$-doping at $\text {Pb}^{+2}$ site) in a *C*-type AFM Mott-insulator $\text {Pb}_2 \text {FeOsO}_6$ ($\text {Pb}_{2-x} \text {Na}_x \text {FeOsO}_6$) DPO results in an HM FiM state, and the holes produced by Na doping resides in the Os 5*d* orbitals^43^. Lv et al., first-principles calculations exhibited that electron doping (i.e., $\text {La}^{+3}$-doping at $\text {Ba}^{+2}$ site) leads a transition from Mott-insulator AFM to HM AFM in $\text {Ba}_{2-x} \text {La}_x \text {MnMoO}_6$ for *x* $\le 1.0$ and then to a metallic AFM state for $\textit{x} = 1.5$ and 2.0^48^. It is also predicted that extra electrons go to the Mo 4*d* orbitals, which are responsible for metallicity in these doped systems. Very recently, Bhandari et al.^47^, theoretically demonstrated that electron doping at B site (i.e., 50% Ni-doping at Cr site) in a FiM Mott-insulator $\text {Ca}_2 \text {CrOsO}_6$ gives rise to a nearly compensated HM state which has potential applications for spintronic devices. In this case, Ni is in a $+$2 state with $3d^8$ configurations and when it is replaced with Cr of charge of $+3$ having a configuration of $3d^3$, therefore, five extra electrons added to the system which results in an HM state.

In this regard, this doping strategy in recently synthesized FiM Mott-insulator LNIO DPO^18^ could be very beneficial for its utilization in spintronic devices because of the low/high energy band gap of 0.20/2.25 eV in the spin-majority/spin-minority channel^30^ with a higher $T_C$ of 207 K among the disorder DPOs. Therefore, we explored a possibility to produce the HM materials via hole-doping engineering, that is, to introduce one 3*d* TM = Cr, Mn, and Fe of impurity ions at one of the B-site atom (i.e., which is also a 3*d* TM site: Ni site) in LNIO system using first-principles electronic structure calculations. Hence, the doping concentration of TM-doped atoms is 50% in the stable solid solution. Therefore, the TM-doped LNIO structures are most likely referred to as an alloy. These potential elements show HM nature having absolute magnetic moments with improved FiM ordering temperature.

## Results and discussion

### Structural proprties

**Figure 1:**
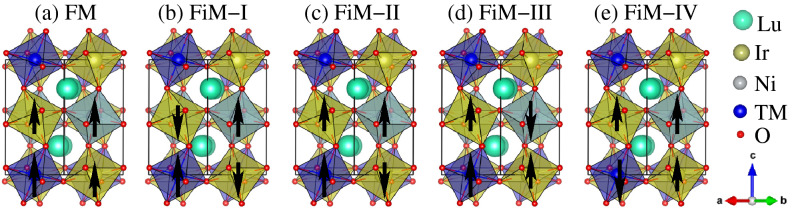
Schematic representation of the double perovskite structures of $\text {Lu}_2 \text {Ni}_{0.5} \text {TM}_{0.5} \text {IrO}_6$ (TM = Cr, Mn, and Fe) in (**a**) ferromagnetic (FM), (**b**) ferrimagnetic (FiM)-I, (**c**) FiM-II, (**d**) FiM-III, and (**e**) FiM-IV spin ordering. The tilted octahedral surroundings of the Ni, TM, and Ir with oxygen atoms are visualized in light grey, light blue, and light green colored polyhedron, respectively. The *a*, *b*, and *c*-axes are along the crystallographic *x*, *y*, and *z*-directions, respectively.

LNIO crystallizes in the monoclinic structure with space group No. 14 ($P2_1/n$) and the experimental lattice parameters are $\textit{a} = 5.21431$, $\textit{b} = 5.63533$, and $\textit{c} = 7.53905$ Å with $\beta = 90.1834^{\circ }$^18^. The Lu, Ni, Ir, O1, O2, and O3 ions occupy the Wyckoff positions 4e(0.9755,0.07858,0.2507), 2c(0.5,0,0.5), 2b(0.5,0,0), 4e(0.1372,0.4484,0.2521), 4e(0.6820,0.3061,0.0592), and 4e(0.1786,0.1880,0.9396), respectively. Our prior DFT calculations^30^ and Feng et al. experiment^18^ exhibit that Mott-insulating FiM state is more energetically stable than that of FM and AFMs structures in pristine LNIO, because AFM superexchange coupling between Ni and Ir are strongly favorable at the diagonals. Moreover, it is found that the next magnetic state close to the FiM ground state is FM^30^, therefore, we considered the FM and different FiM structure for further doping engineering in the present study. The schematic representation of crystal structures of $\text {Lu}_2 \text {Ni}_{0.5} \text {TM}_{0.5} \text {IrO}_6$ in an FM, FiM-I, FiM-II, FiM-III, and FiM-IV spin orderings are shown in Fig. 1a–e, respectively. In an FM spin ordering, Ni, TM, and Ir ions spins are parallel (Fig. 1a), while they are anti-parallel within both in-plane and out-of-plane directions in FiM-I state (Fig. 1b). In the case of FiM-II structure, Ni, TM, and one Ir ion spins are parallel to each other (i.e., ferromagnetically coupled) and the second Ir ion is anti-parallel to all of them as shown in Fig. 1c. For FiM-III spin ordering, TM and both Ir ions spins are anti-align than that of Ni (Fig. 1d), while both Ir and Ni ions spins are aligned with each other than that of TM in FiM-IV structure (Fig. 1e). Here, it is very important to note that only ordered TM-doped structures are considered likewise undoped one. However, a disordered between Ni and TM ions may exist due to a small difference in their size or charge which highly demands its experimental verification.

### Structural stability and magnetic ground state

**Figure 2:**
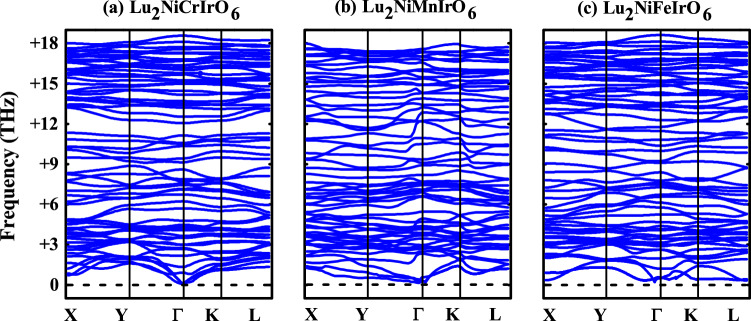
Calculated phonon dispersion band structures for (**a**) Cr-, (**b**) Mn-, and (**c**) Fe-doped $\text {Lu}_2 \text {NiIrO}_6$ double perovskite oxides.

To estimate the relative structural stability of TM-doped LNIO systems, formation energy ($E_f$) in each case is calculated as:

1 $$\begin{aligned} E_f = E_{doped} - E_{undoped} - \mu _{TM} + \mu _{Ni} \end{aligned}$$

where $E_{doped}$ and $E_{undoped}$ refers to the total energy of TM-doped and undoped LNIO DPO, respectively. $\mu _{TM}$ and $\mu _{Ni}$ are the chemical potentials of the TM and Ni atoms, which are the total energies of the most stable low-temperature phases of the bulk TM and Ni ($E_{TM}$ and $E_{Ni}$), respectively. The calculated values of $E_f$ for Cr-, Mn-, and Fe-doped LNIO materials are − 0.52, − 1.21, and − 1.65 eV, respectively (also listed in Table 1). The “−” values of $E_f$ for all TM-doped systems indicate that they are thermodynamically stable and can be easily synthesized. Furthermore, the phonon dispersion calculations have been performed to check the dynamic stability of doped systems. The computed phonon dispersion bands are plotted in Fig. 2a–c for Cr-, Mn, and Fe-doped LNIO structures, respectively. The phonon dispersions show a set of 60 phonon branches owing to the presence of 20 atoms per primitive cell. The higher frequency manifold is mainly attributed to vibrations of oxygen atoms which are dispersive and well separated in the higher energy regime as compared to the atomic species^49^. The absence of the imaginary frequencies in all TM-doped systems, which provides real evidence of the structures dynamic stability. The lower manifold from 0 to $+$6 THz consists of the acoustic modes (24 phonon branches for each case) but it can also be noticed that some of the modes have strongly mixed character, which may lead the systems to metallicity.

**Table 1:** Calculated formation energy ($E_f$) in eV, magnetic energy differences: $\Delta E_1 = E_{FM}-E_{FiM-I}$, $\Delta E_2 = E_{FM}-E_{FiM-II}$, $\Delta E_3 = E_{FM}-E_{FiM-III}$, and $\Delta E_4 = E_{FM}-E_{FiM-IV}$ in meV, FiM ordering temperature ($T_{C1}$) in K, and energy band gap ($E_g$) in eV for $\text {Lu}_2 \text {Ni}_{0.5} \text {TM}_{0.5} \text {IrO}_6$ (TM = Cr, Mn, and Fe) double perovskite oxides.

TM	$E_f$	$\Delta E_1$	$\Delta E_2$	$\Delta E_3$	$\Delta E_4$	$T_{C1}$	$E_g$	$m_{tot.}$/u.c.	$m_{tot.}$/f.u.	$m_{Ni}$	$m_{TM}$	$m_{Ir1}$	$m_{Ir2}$	$m_{O1}$	$m_{O2}$	$m_{O3}$
Undop.	−	28.3	25.5	17.8	17.5	219	0.20	1.90	0.95	1.66	1.66	− 0.54	− 0.54	− 0.01	− 0.03	− 0.02
Cr	− 0.52	37.7	35.5	33.3	31.9	292	HM	4.00	2.00	1.66	2.66	− 0.09	− 0.39	− 0.09	− 0.39	− 0.03
Mn	− 1.21	33.8	31.2	30.5	28.7	261	HM	5.00	2.50	1.69	3.80	− 0.16	− 0.41	− 0.16	− 0.41	− 0.02
Fe	− 1.65	30.5	29.2	28. 9	− 13.8	236	HM	6.00	3.00	1.67	4.11	− 0.05	− 0.42	− 0.06	− 0.42	− 0.09

The “−” and “$+$” signs in $E_f$ and $\Delta E$ show that doped crystal structure and FiM spin ordering are energetically stable, respectively. Moreover, “ HM” in the $E_g$ column represents the half-metallic nature of the system along with total spin moments per unit cell ($m_{tot.}$/u.c.) as well as per formula unit ($m_{tot.}$/f.u.) and partial spin moments on Ni ($m_{Ni}$), TM ($m_{TM}$), Ir ($m_{Ir}$), and O ($m_{O}$) ions.

Next, the energetically favorable magnetic ground state in undoped and doped TM-doped LNIO DPO is examined by comparing the total energies of FM with different FiM structures as $\Delta E_1/\Delta E_2/\Delta E_3/\Delta E_4 = E_{FM}-E_{FiM-I}/E_{FM}-E_{FiM-II}/E_{FM}-E_{FiM-III}/E_{FM}-E_{FiM-IV}$. The computed values of $\Delta E_1$, $\Delta E_2$, $\Delta E_3$, and $\Delta E_4$ are listed in Table 1. The “−” and “$+$” signs of $\Delta E$ means that FM and FiM spin ordering are more energetically stable, respectively. Our calculations show that FiM-I spin ordering in the undoped and all doped structures are energetically favorable (i.e., $\Delta E_1$ is the ground state in each case) than that of FM and remaining FiM structures. This means that Ni and TM ion spins want to remain parallel with each other at the diagonal site, but prevail anti-parallel with Ir in both in-plane and out-of-plane. From $\Delta E_1$, the FiM ordering temperature $T_{C1}$ in each doped case is computed as “$\frac{2/3 \cdot \Delta E}{k_B}$”, where $\text {k}_B$ is the Boltzmann constant. The calculated $T_{C1}$ for undoped, Cr, Mn, and Fe-doped LNIO materials are 219, 369, 339, and 360 K (also listed in Table 1) corresponds to $\Delta E_1$ in each case, respectively. It is also important to note that our computed $T_{C}$ of 219 K in undoped LNIO material is very close to the experimentally observed value of 207 K^18^. Interestingly, present calculations predicted that $T_C$ enhanced when one of the Ni ions is replaced with TM having the highest $T_{C1} = 292$ K for Cr-doped material. This means that the structural distortion increases with the TM-doping as compared to the undoped one. As, FiM-I spin ordering is the energetically favorable magnetic ground state in the undoped and all TM-doped LNIO structures, therefore, for further investigations only FiM-I magnetic structure is taken into account in all cases.

### Octahedral distortion

**Figure 3:**
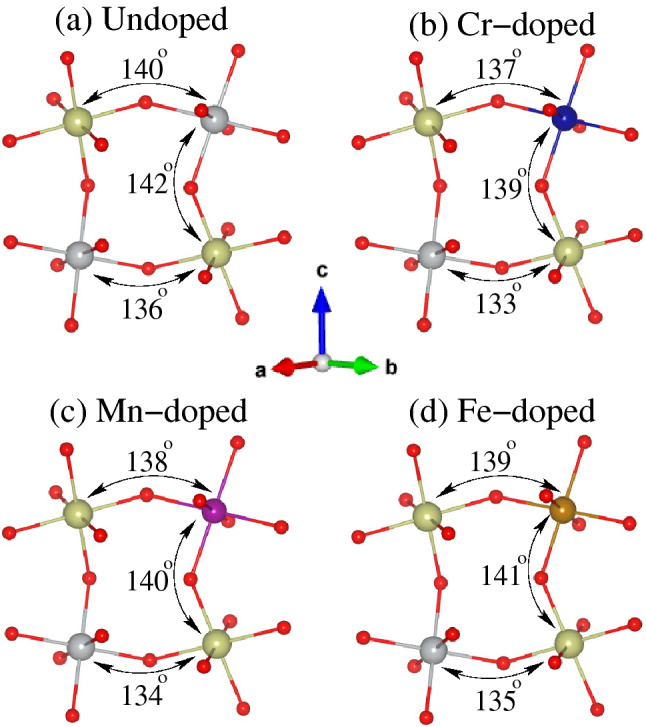
A crystallographic view of the crystal structure of (**a**) undoped, (**b**) Cr-doped, (**c**) Mn-doped, and Fe-doped $\text {Lu}_2 \text {NiIrO}_6$ double perovskite oxides along with DFT relaxed three peculiar Ni(TM)-O-Ir bond angles. The *a*, *b*, and *c*-axes are along the crystallographic *x*, *y*, and *z*-directions, respectively.

It is previously established that deviation of 3*d*-O-5*d* bond angles from the perfect geometry of the high cubic symmetry structure ($180^{\circ }$) in a distorted DPOs lead to a strong AFM superexchange coupling between magnetic ions, which results in a FiM ordering^46,50–54^ and large structural distortions enhance the FiM ordering temperature^55–57^. Therefore, we displayed the DFT relaxed three peculiar TM-O-Ir bond angles on $\text {TMO}_6$ and $\text {IrO}_6$ octahedron for undoped, Cr-doped, Mn-doped, and Fe-doped LNIO materials in Fig. 3a–d, respectively. The calculated Ni-O1-Ir/Ni-O2-Ir/Ni-O3-Ir bond angles of $136^{\circ }/142^{\circ }/140^{\circ }$ in undoped LNIO (see Fig. 3a) are in a good agreement with the experimentally observed values of $135.4^{\circ }/142.5^{\circ }/140.8^{\circ }$^18^. Surprisingly, our results show that the substitution of smaller TM cations into Ni-site produced a significant structural distortion which further affects the electronic and magnetic properties. As the Cr-doped LNIO system exhibits large structural distortions (i.e., smaller bond angles) than that of undoped and other doped systems, compare the bond angles magnitudes in Fig. 3b with that of Fig. 3a,c,d, respectively. This usually demands a higher energy difference between the different magnetic structures, which results in a higher $T_C$ as displayed in Table 1.

### Electronic properties

**Figure 4:**
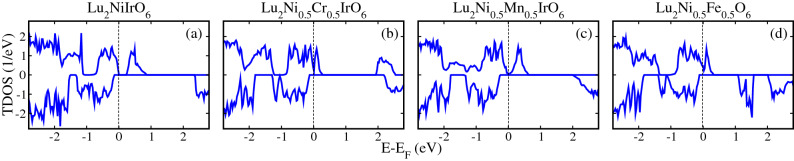
Calculated spin-polarized total density of states (TDOS) within a $\text {GGA}+U$ method for (**a**) undoped $\text {Lu}_2 \text {NiIrO}_6$, (**b**) Cr-doped $\text {Lu}_2 \text {NiIrO}_6$ ($\text {Lu}_2 \text {Ni}_{0.5} \text {Cr}_{0.5} \text {IrO}_6$), (**c**) Mn-doped $\text {Lu}_2 \text {NiIrO}_6$ ($\text {Lu}_2 \text {Ni}_{0.5} \text {Mn}_{0.5} \text {IrO}_6$), and (**d**) Fe-doped $\text {Lu}_2 \text {NiIrO}_6$ ($\text {Lu}_2 \text {Ni}_{0.5} \text {Fe}_{0.5} \text {IrO}_6$) double perovskite oxides in a FiM-I spin ordering. The verticle dotted line represents the Fermi level in each DOS case.

To explicitly display the TM-doping impact on the electronic properties of LNIO DPO, we first produced the spin-polarized total density of states (TDOS) for undoped LNIO material in Fig. 4a to provide a frame of reference. One can see that system exhibits an insulating behavior with an $E_g$ of 0.20/2.25 eV in the spin-majority/spin-minority channel, which is in good agreement with experimental^18^ and previous theoretical work^30^. Next, we studied the TM-doping influence on the electronic structure of LNIO which exhibits substantial changes. For this, spin-polarized TDOS for Cr, Mn, and Fe-doped LNIO systems are plotted in Fig. 4b–d, respectively, where all the doped materials showing the HM behavior in which the spin-majority channel is conductor while the spin-minority channel is an insulator.

**Figure 5:**
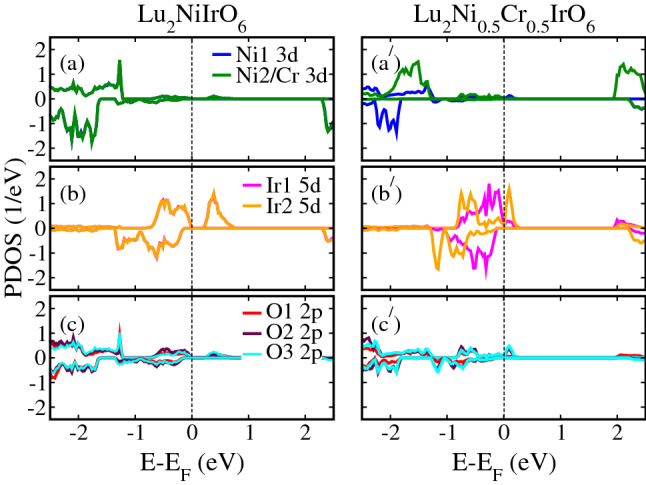
Calculated spin-polarized partial density of states (PDOS) within a $\text {GGA}+U$ method for (**a**/**a′**) Ni1 and Ni2/Cr 3*d*, (**b**/**b′**) Ir1/Ir2 5*d*, and (**c**/**c′**) O1/O2/O3 2*p* orbitals in undoped $\text {Lu}_2 \text {NiIrO}_6$ (left column) and Cr-doped $\text {Lu}_2 \text {NiIrO}_6$ ($\text {Lu}_2 \text {Ni}_{0.5} \text {Cr}_{0.5} \text {IrO}_6$: right column) double perovskite oxides.

To elucidate the origin of the metallic electronic states in these doped systems, we plotted the spin-polarized partial density of states (PDOS) projected on the Ni/Cr 3*d*, Ir 5*d*, and O 2*p* orbitals in undoped LNIO (left column) and Cr-doped LNIO ($\text {Lu}_2 \text {Ni}_{0.5} \text {Cr}_{0.5} \text {IrO}_6$: right column) DPO in Fig. 5, for example. Our calculations clearly show that both 3*d* states of Ni ions stay away from the Fermi level ($E_F$) in undoped LNIO material (see Fig. 5a), while Ir 5*d* states grow around $E_F$ in both valence and conductions bands (Fig. 5b) with small contributions from O 2*p* states (Fig. 5c). Furthermore, the PDOS analysis in undoped LNIO material indicates that Ni1 and Ni2 3*d* spin-polarized states are overlapped which means that both ions are showing equivalent contributions to the electronic structure. A similar trend of PDOS can also be seen for Ir and oxygen ions. In the case of Cr-doped LNIO ($\text {Lu}_2 \text {Ni}_{0.5} \text {Cr}_{0.5} \text {IrO}_6$) material, Ni/Cr 3*d* states lie very below and above the $E_F$ in the valence and conduction bands (Fig. 5$\text {a}^\prime$), respectively and not contributing to the metallicity as found in the case of undoped one. Interestingly, Ir2 5*d* states substantially shift from valence to conduction band by crossing $E_F$ in the spin majority channel and are primarily responsible for metallicity with a significant contribution from Ir1 5*d* states (see Fig. 5$\text {b}^\prime$). A small contribution of O 2*p* states to the metallicity in the spin majority channel is also evident as displayed in Fig. 5$\text {c}^\prime$. In contrast, an insulating behavior is established in the spin-minority channel for each PDOS as found in the case of undoped one. Hence, with the metallic electronic state in the spin-majority channel and an insulating in the spin-minority channel, the $\text {Lu}_2 \text {Ni}_{0.5} \text {Cr}_{0.5} \text {IrO}_6$ material is predicted to be an HM. Here it is very important to note that Ir1 PDOS tends towards degeneracy, which means that it is close to non spin-polarized state having an almost negligible magnetic moment (discuss below). A similar PDOS behavior is also found in the case of Mn- and Fe-doped systems ($\text {Lu}_2 \text {Ni}_{0.5} \text {Mn}_{0.5} \text {IrO}_6$ and $\text {Lu}_2 \text {Ni}_{0.5} \text {Fe}_{0.5} \text {IrO}_6$) as shown in Figs. 1S and 2S of the Supporting Information (SI), respectively.

For a more deep understanding of the origin of metallic electronic states near $E_F$, we produced the orbital-resolved PDOS on Ni/Cr/Ir 3*d*/3*d*/5*d* orbitals in undoped LNIO (left column) and Cr-doped LNIO ($\text {Lu}_2 \text {Ni}_{0.5} \text {Cr}_{0.5} \text {IrO}_6$: right column) systems in Fig. 3S of the SI. It is very well established that the crystal field splits the d orbitals into low energy triply $t_{2g}$ ($d_{xy}$, $d_{xz}$, and $d_{yz}$) and high energy doubly $e_g$ ($d_{x^2-y^2}$ and $d_{3z^2-r^2}$) states. Moreover, the tetrahedron distortion from perfect order reduces the $O_h$ symmetry to $D_{4h}$ and finally lifts the orbital degeneracy. Hence, the $t_{2g}$ further splits into singlet $d_{xy}$, $d_{xz}$, and $d_{yz}$ non-degenerate states. Similarly, the $e_g$ is also divided into singlet $d_{x^2-y^2}$ and $d_{3z^2-r^2}$ non-degenerate states. From Fig. 3S(a) and 3S(b) of the SI, one can see that Ni1 and Ni2 3*d* orbitals lie away from the $E_F$ in undoped case, respectively. However, the admixture of Ir1 and Ir2 5*d* orbitals are dominant in both valence and conduction bands, see Fig. 3S(c,d), respectively. It is also very clear that a band gap exists between Ir 5*d* orbitals in undoped LNIO structure, which also confirms the Mott-insulating state of the system. Similarly, Ni and Cr 3d orbitals are almost showing the negligible contributions around $E_F$ in Cr-doped LNIO system as display in the Fig. 3S($\text {a}^\prime$) and 3S($\text {b}^\prime$) of the SI. Interestingly, the admixture of Ir1 (Fig. 3S($\text {c}^\prime$)) and Ir2 (Fig. 3S($\text {d}^\prime$)) 5*d* orbitals in the spin-majority channel are crossing the $E_F$ from the valence to conduction bands and are responsible for metallicity. However, the spin minority channels are exhibiting the insulating behavior, which results in the HM state of the system in Cr-doped LNIO system.

**Figure 6:**
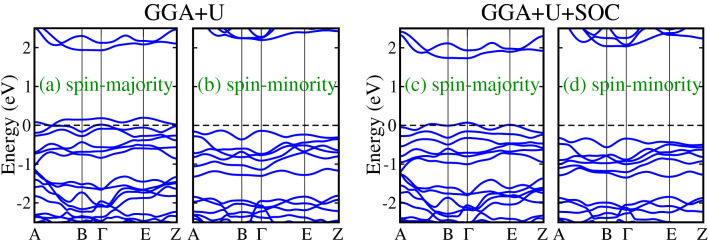
Calculated spin-polarized (**a**/**c**) spin-majority and (**b**/**d**) spin-minority band structures within $\text {GGA}+U/\text {GGA}+U+\text {SOC}$ method in Cr-doped $\text {Lu}_2 \text {NiIrO}_6$ ($\text {Lu}_2 \text {Ni}_{0.5} \text {Cr}_{0.5} \text {IrO}_6$) double perovskite oxide.

For direct observation of the metallic electronic states in doped systems, we plotted the spin-polarized band structures along with the high symmetry directions of the monoclinic Brillouin zone for $\text {Lu}_2 \text {Ni}_{0.5} \text {Cr}_{0.5} \text {IrO}_6$ material within both $\text {GGA}+U$ (left column) and $\text {GGA}+U+\text {SOC}$ (right column) methods in Fig. 6, for instance. One can see that in the spin-majority channel, bands grow at the $E_F$ and exhibits metallic behavior as displayed in Fig. 6a within $\text {GGA}+U$ scheme. On the other hand, the spin-minority channel shows an insulating nature having an energy gap of 2.32 eV. Hence, the spin majority bands are partially occupied, therefore, an HM state is formed which also confirms the calculated TDOS in Fig. 4b. Contrarily, a considerable downshift in the spin-majority and spin-minority bands are evident with the inclusion of SOC as compared to $\text {GGA}+U$ method, compare the Fig. 6c,d with Fig. 6a,b, respectively. However, still a few spin-majority bands are crossing the $E_F$ from valence to conduction channel for $\text {GGA}+U+\text {SOC}$ calculations (see Fig. 6c), while spin-minority channel remains insulator (see Fig. 6d). Hence one can conclude that despite the down shift of the bands within the $\text {GGA}+U+\text {SOC}$ method, the HM state of the doped system remains the same as found in the case of $\text {GGA}+U$ (without SOC) scheme.

**Figure 7:**
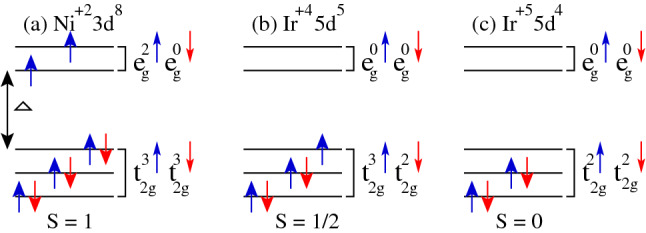
Schematic representation of (**a**) $\text {Ni}^{+2}\,3d^8$, (**b**) $\text {Ir}^{+4}\,5d^5$, and (**c**) $\text {Ir}^{+5}\,5d^4$ orbital configurations.

To understand the physical mechanism behind the metallic behavior in these doped materials, we analyze the electronic configurations of ions in each case. In undoped LNIO, Ni is in $+$2 state with a $3d^8$ configuration which indicates that $t_{2g}$ and $e_g$ states are fully and partially occupied, respectively. This means that three electrons lie in the spin-majority and three in the spin-minority channels of $t_{2g}$ states (i.e., $t_{2g}^3\uparrow$ and $t_{2g}^3\downarrow$). The remaining two electrons partially filled the $e_g$ states of the spin-majority channel as $e_{g}^2\uparrow e_{g}^0\downarrow$. Thus, $t_{2g}$ states completely remain in the valence band, while two up-filled and two down(dn)-empty states of $e_g$ reside in the valence and conduction bands, respectively. Similarly, Ir is in a $+4$ state having a configuration of $5d^5$ in which $t_{2g}$ and $e_g$ states are partially and empty, respectively. Five electrons of Ir ion are distributed as: three and two electrons lie in the spin-majority and spin-minority channels of $t_{2g}$ states ($t_{2g}^3\uparrow t_{2g}^2\downarrow$), respectively. Hence, five (i.e., three up and two dn) states of Ir $t_{2g}$ reside in the valence band and remaining shifts in the conduction region. On the other hand, all the Ir $e_g$ states remain in the conduction band. The schematic representation of electronic configurations for $\text {Ni}^{+2}\,3d^8$ and $\text {Ir}^{+4}\,5d^5$ orbitals are shown in Fig. 7a,b, respectively.

For $\text {Lu}_2 \text {Ni}_{0.5} \text {Cr}_{0.5} \text {IrO}_6$ doped material, Cr is in $+$3 state with a $3d^3$ configuration and produces a deficiency of five electrons when substituted at $\text {Ni}^{+2}\,3d^8$ sites. Hence, the hole was created due to a lack of electrons, therefore, $E_F$ shifts towards lower energies, and few bands crossing the $E_F$ from the valence to the conduction band. This results in a metallic state in the spin-majority channel, while a large band gap still exists in the spin-minority channel which leads the system into an HM state (see Fig. 4b). Similarly, Mn lies in $+$3 state having $3d^4$ configurations and the system lacks four electrons when it is replaced with $\text {Ni}^{+2}\,3d^8$ ions. Hence, $e_g$ states in the spin-majority channel will become fully unoccupied and $E_F$ shifts towards lower energies. Thus, few bands near $E_F$ in the spin-majority channel crossing the $E_F$ which result in a metallicity. On the other hand, the spin-minority channel remains insulator, which leads the system into an HM state (see Fig. 4c). Finally, Fe is also in $+$3 state with a configuration of $3d^5$ which produces a deficiency of three electrons when it replaced with $\text {Ni}^{+2}\,3d^8$ ion, which drags the system into HM state (see Fig. 4d) as discussed above.

### Magnetism

Next, we examined the calculated total/atom-resolved partial spin magnetic moments in undoped and TM-doped LNIO systems. As allocated in Table 1, the calculated total spin magnetic moments of 1.90(0.95), 4.0(2.0), 5.0(2.5), and 6.0(3.0) $\mu _B$ per unit cell(per formula unit) for undoped, Cr-, Mn-, and Fe-doped LNIO materials, respectively. The integral moments per unit cell in all three doped systems affirm the HM state. For the undoped system, the calculated $m_{tot.}$ of 0.95 $\mu _B$/f.u. is almost two times of the experimentally observed value of 0.52 $\mu _B$/f.u.^18^. This overestimation is because antisite disorder is not taken into the account in the calculations. As it is theoretically found that when antisite disorder was considered in LNIO system^18^, the $m_{tot.}$ was 0.78 $\mu _B$/f.u. which is impending to the experimentally observed value of 0.52 $\mu _B$/f.u. at a very low temperature of 5 K. This shows that Ni and Ir ions remain in $+$2 and $+4$ states having electronic configurations of $t_{2g}^3\uparrow t_{2g}^3\downarrow \,e_g^2\uparrow \,e_g^0\downarrow$ and $t_{2g}^3\uparrow t_{2g}^2\downarrow \,e_g^0\uparrow \,e_g^0\downarrow$ with spin states of $\text {S} = 1$ and $\text {S} = 1/2$, respectively. Moreover, the computed partial spin magnetic moments on Ni and Ir ions are 1.66 and − 0.54 $\mu _B$, respectively which means that the Ni ion mainly contributing to $m_{tot.}$. The “−” sign in the Ir moment indicates that its spin is anti-align to that of the Ni. This confirms the strong AFM superexchange coupling between $\text {Ni}^{+2}$ and $\text {Ir}^{+4}$ ions via oxygen ($\text {Ni}^{+2}$–$\text {O}^{-2}$–$\text {Ir}^{+4}$) which results in a FiM ordering.

In the case of doped materials, Ni moment magnitude almost remains the same ($\sim 1.66\,\mu _B$) as found in the case of undoped one (Table 1), which means that it remains in a $+2$ state. However, a strong reduction in the Ir1 spin magnetic moment is found as compared to the undoped one along with a small decrease in the Ir2 moment is also predicted. The computed spin moments on the Ir1/Ir2 are − 0.54/− 0.54, − 0.09/− 0.39, − 0.16/− 0.41, and − 0.05/− 0.42 in undoped LNIO, doped $\text {Lu}_2 \text {Ni}_{0.5} \text {Cr}_{0.5} \text {IrO}_6$, $\text {Lu}_2 \text {Ni}_{0.5} \text {Mn}_{0.5} \text {IrO}_6$, and $\text {Lu}_2 \text {Ni}_{0.5} \text {Fe}_{0.5} \text {IrO}_6$, respectively. This shows that Ir ion lies in a mixed-valence state of $+4$ and $+4/+5$ with the configurations of $5d^5$ ($t_{2g}^3\uparrow t_{2g}^2\downarrow$) and $5d^5/5d^4$ ($t_{2g}^3\uparrow t_{2g}^2\downarrow /t_{2g}^2\uparrow t_{2g}^2\downarrow$) in undoped and all doped structures, respectively. The schematic representation of Ir in $+5$ state having a configuration of $5d^4$ ($t_{2g}^2\uparrow t_{2g}^2\downarrow$) with S = 0 is shown in Fig. 7c. The individual spin magnetic moments on Cr, Mn, and Fe ions are 2.66, 3.80, and 4.11 $\mu _B$, respectively. Besides the spin moments on the TM and Ir ion sites, the oxygen ions at non-equivalent sites are also spin-polarized (see Table 1) and a sizable spin moment of $\sim \,-$0.40 $\mu _B$ arises on the O2 atom in each doped case (see Table 1). Because, a strong hybridization between TM/Ir and oxygen atoms, results in a partial charge transfer from TM (i.e., Cr, Mn, and Fe) and Ir ions to oxygen.

**Figure 8:**
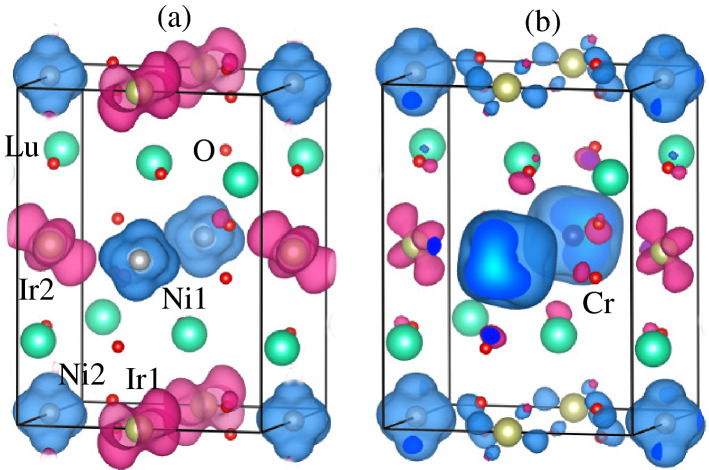
Spin-magnetization density isosurface plots for (**a**) undoped and (**b**) Cr-doped $\text {Lu}_2 \text {NiIrO}_6$ ($\text {Lu}_2 \text {Ni}_{0.5} \text {Cr}_{0.5} \text {IrO}_6$) materials at the same iso-value of $\pm 0.04$ *e*/Å$^3$. The light blue and light pink colors represent the spin up and spin down states, respectively.

To understand the charge transfer mechanism, we plotted the spin-magnetization density isosurface plots for undoped and Cr-doped ($\text {Lu}_2 \text {Ni}_{0.5} \text {Cr}_{0.5} \text {IrO}_6$) materials in Fig. 8a,b, respectively. As one can see in the undoped case (Fig. 8a), Ni 3*d* and Ir 5*d* orbitals are primarily contributing to the spin density. It is a well-known fact that Ni is in $+$2 state with a $d^8$ configuration in LNIO. Thus, the $t_{2g}$ ($d_{xy}$, $d_{yz}$, and $d_{xz}$) states are fully occupied by maintaining six electrons, while the remaining two unpaired electrons lie in the $e_g$ ($d_{x^2-y^2}$ and $d_{3z^2-r^2}$) states. Therefore, the $e_g$ orbital characteristics are visible in the isosurfaces of the Ni ions in both undoped and Cr-doped LNIO materials as displayed in Fig. 8a,b, respectively. Moreover, very small spin densities have also appeared on the oxygen atoms. Because of a small charge transfer on the oxygen ions due to superexchange coupling between $\text {Ni}^{+2}$ and $\text {Ir}^{+4}$ ions via oxygen ($\text {Ni}^{+2}$-$\text {O}^{-2}$-$\text {Ir}^{+4}$). Interestingly, substantial changes occur in the magnitude of spin densities when one of the Ni ions is replaced with Cr, see Fig. 8b. The most striking feature of the present calculations is that spin-density around Ir1 ion is almost disappeared, while a small decrease in Ir2 is evident which confirms the calculated spin magnetic moments on Ir ions in Cr-doped LNIO system in Table 1. Moreover, the spin density nature of Cr ion is due to the admixture of $d_{xy}$, $d_{yz}$, and $d_{xz}$ (i.e., $t_{2g}$) orbitals character. Besides, a substantial amount of spin density arises on the oxygen ions in Cr-doped (Fig. 8b) system than that of undoped one (Fig. 8a), because extra charge transfer from Ni/Cr and Ir ions to oxygen due to strong hybridization between them.

## Conclusion

Employing non-degenerate DFT calculations, the electronic and magnetic properties of undoped and transition metal (TM) = Cr, Mn, and Fe-doped $\text {Lu}_2 \text {NiIrO}_6$ double perovskite oxides are investigated, where TM ions having 50% concentration are substituted at Ni-site. We found that the undoped material is a ferrimagnetic (FiM) Mott-insulator, while all the doped structures exhibit half-metallic FiM behavior. The metallic electronic states in the spin majority channels mainly belong to the admixture of Ir 5*d* orbitals in each case. It is also established that Ir ion lies in a mixed-valence state of $+4$ and $+5$ in all doped systems with configurations of $5d^5$ ($t_{2g}^3\uparrow t_{2g}^2\downarrow$) and $5d^4$ ($t_{2g}^2\uparrow t_{2g}^2\downarrow$), respectively, which results in a strong reduction of magnetic moment on the Ir $5d^4$ ion. Interestingly, our calculations revealed that structural distortion enhanced when one of the Ni ions is replaced with TM, and maximum deviation from the perfect cubic symmetry is obtained in the case of the Cr-doped $\text {Lu}_2 \text {NiIrO}_6$ material, which further optimize the FiM ordering temperature. Therefore, $\text {Lu}_2 \text {Ni}_{0.5} \text {Cr}_{0.5} \text {IrO}_6$ system exhibits a higher FiM ordering temperature of 292 K as compared to undoped and other doped systems. Hence, these doped materials show promise of their probable feasible applications in hard magnetic memory devices.

## Computational methods

First-principles electronic structure calculations based on DFT were performed using a full-potential linearized augmented plane wave method as implemented in the WIEN2K code^58^. The exchange-correlation functional which is parameterized by generalized gradient approximation (GGA)^59^ plus on-site Coulomb interaction (GGA+*U*) approach was employed with *U* = 3.85, 3.5, 4.0, 5.0, 5.1, and 2.8 eV for Lu 4*f*, Cr 3*d*, Mn 3*d*, Fe 3*d*, Ni 3*d*, and Ir 5*d* states, respectively^60^. In all cases, the spin non-degenerate version of the GGA is utilized for both with and without SOC calculations. The relativistic (i.e., SOC) effects attributed with spin-magnetization align along the [100], [010], [001], [110], [101], [011], and [111] directions and found that [001] axis is the easy axis. For the wave function expansion inside the atomic spheres, a maximum value of $l_{max} = 12$ is chosen and the plane-wave cutoff is set to $R_{mt}\times K_{max}= 7$ with $G_{max}= 24$. A $7\times 7\times 9$ *k*-space grid with 128 points within the irreducible wedge of the Brillouin zone is found to be well converged. Along with this, full relaxation of the atomic positions by minimizing the atomic forces up to 2 mRy/a.u. is taken into account in each case. Self-consistency is assumed for a total energy convergence of less than $10^{-5}$ Ry.

## Supplementary Information

Supplementary Information.
