# Supplementary material for: Insulator-to-half metal transition and enhancement of structural distortions in Lu2NiIrO6 double perovskite oxide via hole-doping
Source: Sci Rep. 2021 Jan 13;11:1240. doi: 10.1038/s41598-020-80265-6 (PMC7806915; doi:10.1038/s41598-020-80265-6)
Supplement: Supplementary file 1 — Supplementary Information. [file 41598_2020_80265_MOESM1_ESM.pdf]

Supporting Information

Insulator-to-Half Metal Transition and Enhancement of Structural Distortions in Lu_2NiIrO_6 Double Perovskite Oxide via Hole-Doping

Safdar Nazir*

Department of Physics, University of Sargodha, Sargodha Campus, 40100 Sargodha, Pakistan.

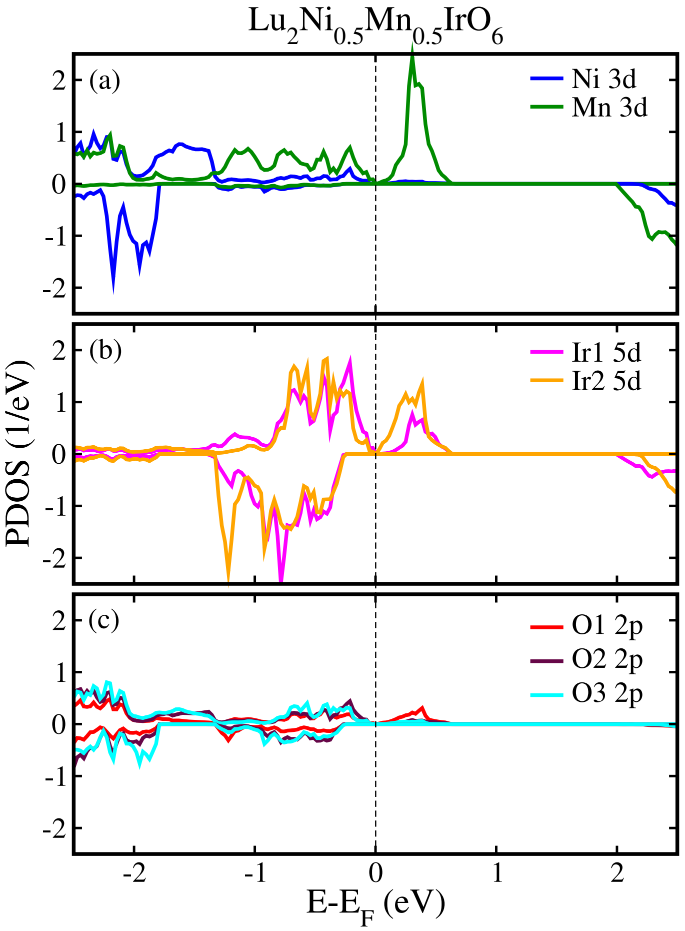

FIG. 1S: Calculated spin-polarized partial density of staes (PDOS) within a GGA+U method for (a) Ni1/Mn 3d, (b) Ir1/Ir2 5d, and (c) O1/O2/O3 2p states in Mn-doped Lu_2NiIrO_6 ($Lu_2Ni_{0.5}Mn_{0.5}IrO_6$) double perovskite oxide.

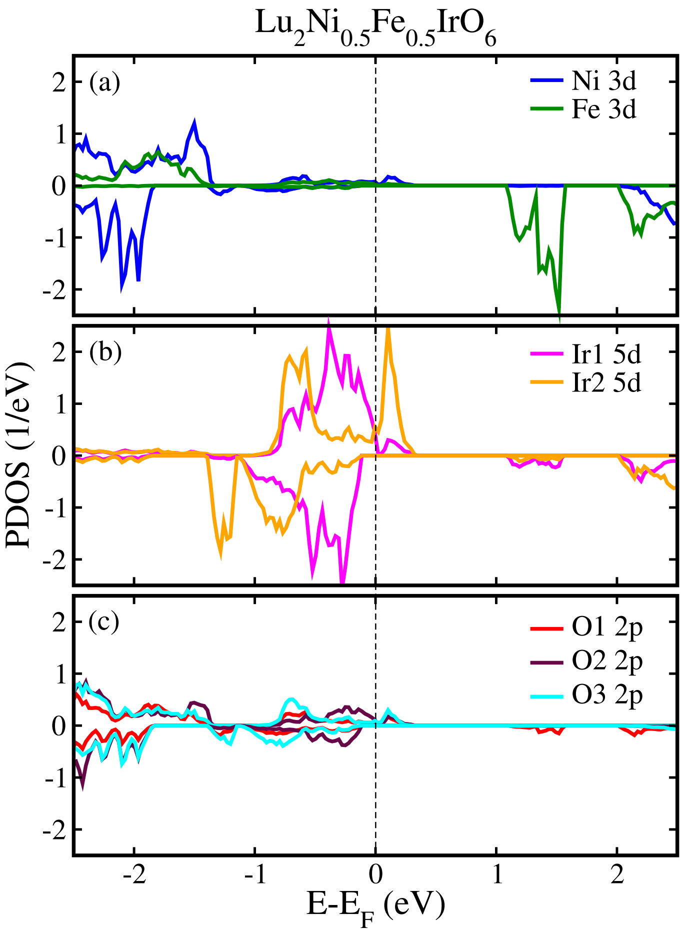

FIG. 2S: Calculated spin-polarized partial density of staes (PDOS) within a GGA+U method for (a) Ni1/Fe $3d$, (b) Ir1/Ir2 $5d$, and (c) O1/O2/O3 $2p$ states in Fe-doped Lu_2NiIrO_6 ($Lu_2Ni_{0.5}Fe_{0.5}IrO_6$) double perovskite oxide.

---

* Electronic address: safdar.nazir@uos.edu.pk,Tel:+92-334-971-9060

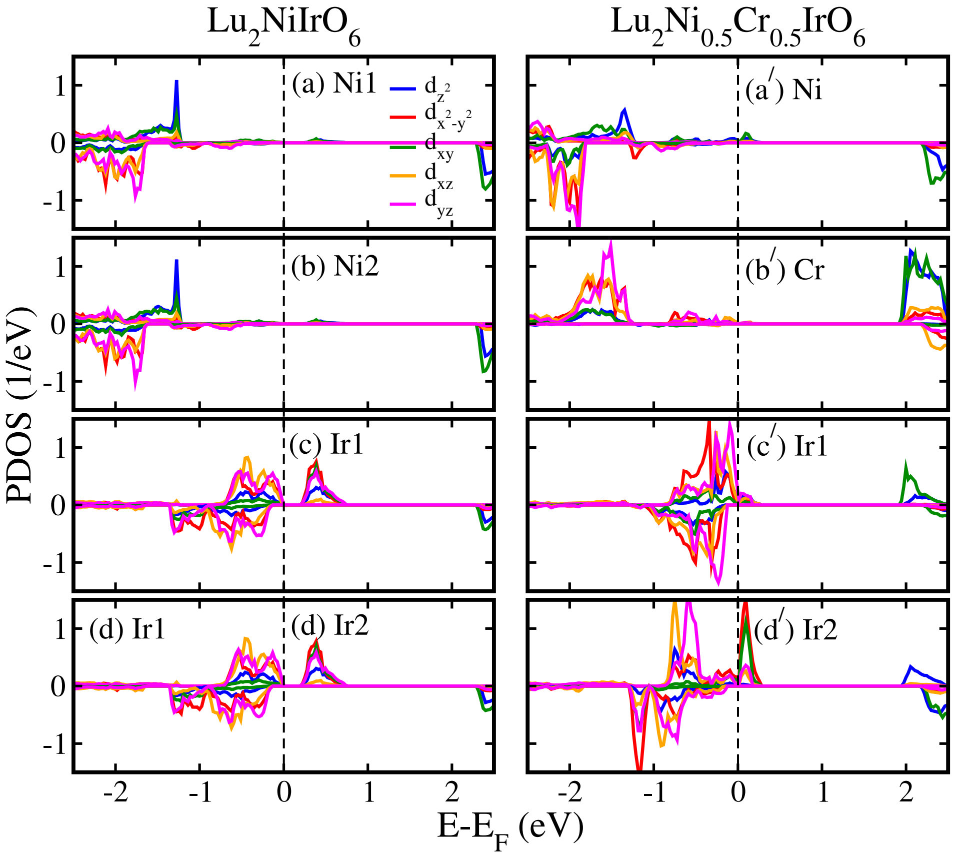

FIG. 3S: Calculated spin-polarized within a GGA+U method for (a and a′) Ni1 and Ni 3d, (b and b′) Ni2 and Cr 3d, (c and c′) Ir1 and Ir1 5d, and (d and d′) Ir2/Ir2 5d orbital resolved partial density of states (PDOS) in undoped Lu_2NiIrO_6 (left column) and Cr-doped Lu_2NiIrO_6 ($Lu_2Ni_{0.5}Cr_{0.5}IrO_6$: right column) double perovskite oxides.
